# A Comparison of Different Surgical Treatments for Complex Anal Fistula: A Systematic Review

**DOI:** 10.7759/cureus.28289

**Published:** 2022-08-23

**Authors:** Anam Zahra, Jyothirmai Malla, Ramaneshwar Selvaraj, Ravneet K Dhanoa, Sathish Venugopal, Shoukrie I Shoukrie, Tharun Y Selvamani, Ranim K Hamouda, Pousette Hamid

**Affiliations:** 1 Surgery, California Institute of Behavioral Neurosciences & Psychology, Fairfield, USA; 2 Internal Medicine, California Institute of Behavioral Neurosciences & Psychology, Fairfield, USA; 3 Neurology, California Institute of Behavioral Neurosciences & Psychology, Fairfield, USA; 4 Orthopedics and Traumatology, California Institute of Behavioral Neurosciences & Psychology, Fairfield, USA

**Keywords:** fecal incontinence, endorectal advancement flap, draining seton, high cryptoglandular perianal fistula, rectal fistula

## Abstract

Fistula-in-ano is a common proctological condition that primarily affects younger people and leads to chronic morbidity. An anal fistula is divided into simple and complex fistulas. A complex fistula is a challenging problem due to higher recurrence rates and incontinence associated with surgery. Many new methods have been developed for the closure of complex fistula-in-ano, but there is no single best method. The aim of this study is to identify a superior surgical technique for treating complex/high cryptoglandular perianal fistulas (HCPFs). A literature search was done using PubMed and Google Scholar for the period of 2012-2021. Articles that contain surgical treatment for complex anal fistula in the English language published in the last 10 years were included. The types of studies included were randomized controlled trials (RCTs), meta-analyses, systematic reviews, cohort studies, and traditional reviews. Articles excluded were those done more than 10 years ago, in other languages, and containing simple fistula management only. Nine studies were included in the review; a systematic review and meta-analysis concluded that no single method is effective. The ligation of the intersphincteric fistula tract (LIFT) procedure seems to be a promising and effective technique as it has a low rate of fecal incontinence as compared to other methods. Biological techniques give variable success rates so does fistula plug (FP). Mucosal advancement flap (MAF) and rerouting seton give good results according to one study. Fistula plug gives variable results and is not a preferred method.

Ligation of the intersphincteric fistula tract (LIFT) seems to be a promising new technique for complex anal fistulas, but the data available is not enough to determine the best method. More randomized trials are required to compare traditional techniques and emerging new biological methods to see the best technique available.

## Introduction and background

Fistula-in-ano is a common condition that primarily affects young people and leads to chronic morbidity. A statistical analysis using a large population database in the UK has shown that the incidence of anal fistula is 1.69 cases per 10,000 individuals [1]. This was also evidenced by other relevant studies [2]. The age range for developing anal fistula is 30-40 years old, and the incidence in males is higher than in females [3]. Park’s classification divides fistula into four different types: intersphincteric, transsphincteric, suprasphincteric, and extrasphincteric. Anal fistula is also divided into simple and complex fistula. According to the classification of the American Society of Colon and Rectal Surgeons (ASCRS), simple types include low transsphincteric and intersphincteric fistulas; they account for less than 30% of the sphincter complex. However, a complex anal fistula is a transsphincteric fistula that includes more than 30% of the sphincter complex. Anal fistulas related to chronic diarrhea, inflammatory bowel disease, radiation, malignancy, or preexisting fecal incontinence are all characterized as complex fistulas.

The aim of surgery in complex anal fistula is to prevent recurrence and avoid incontinence. Different sphincter-sparing techniques were described with variable results including seton, advancement flap, ligation of the intersphincteric fistula tract (LIFT), fistula plug (FP), Fistula-tract Laser Closure (FiLaC), and video-assisted anal fistula treatment (VAAFT). There is no single best treatment for anal fistula yet. Over the past two decades and even in recent years, many new techniques have been developed to treat these fistulas. The mucosal advancement flap (MAF) is one of the most well-known and oldest techniques leading to long-term closure rates of 0%-75% [4-7]. Fibrin glue (FG) was first introduced in the 1990s with the aim of reducing recurrence [8,9]. In 2006, anal fistula plugs (AFP) were introduced and were rigorously investigated after that [10].

In 2007, Rojanasakul et al. introduced the ligation of the intersphincteric fistula tract (LIFT) [11], which was considered a breakthrough in the management of perianal fistulas. Ligation and excision of the intersphincteric tract (LIFT) can close the entrance for fecal particles into the fistula tract and, at the same time, eliminate the intersphincteric septic nidus and promote healing [11]. LIFT does not divide the anal sphincters and does not cause any incontinence.

Some new techniques that use biological products such as fistula plugs, stem cells, and fibrin glue to reduce the risk of incontinence and decrease recurrence are also being developed. These techniques improve outcomes but are not thoroughly applied yet, and a lesser number of studies are available, so their benefit over the traditional techniques has not been proven.

Currently, there is no single best surgical technique for high cryptoglandular perianal fistulas. The goal of our review is to identify a superior surgical technique for treating complex perianal fistulas.

## Review

Methods

Search Strategy

A systematic review was done according to Preferred Reporting Items for Systematic Reviews and Meta-Analyses (PRISMA) 2020 guidelines [12]. A literature search was done using PubMed and Google Scholar for the period of 2012-2021 (i.e., the last 10 years) using the following keywords: anal fistula (“Rectal Fistula” (Majr)) AND (“Rectal Fistula/anatomy and histology” (Majr) OR “Rectal Fistula/pathology” (Majr) OR “Rectal Fistula/surgery” (Majr)) AND fecal incontinence OR high transsphincteric OR suprasphincteric OR extrasphincteric, recurrent and horseshoe fistulas OR multiple tracks OR anterior lying tracks in female patients OR associated with IBD OR radiation OR pre-existing incontinence OR chronic diarrhea (“Fecal Incontinence/pathology” (MeSH) OR “Fecal Incontinence/radiotherapy” (MeSH) OR “Fecal Incontinence/surgery” (MeSH)) AND (“Fecal Incontinence/pathology” (Majr)) AND Endorectal advancement flap OR Draining seton OR anal fistula plug OR debridement and cauterization OR Permacol injection. Additionally, references from retrieved articles were searched for new relevant sources.

Inclusion Criteria

We included articles containing treatments for complex anal fistula, both surgical and alternative methods, in the English language published in the last 10 years. The types of studies included were randomized controlled clinical trials (RCTs), systematic and traditional reviews, meta-analyses, and cohort studies.

Exclusion Criteria

Articles excluded were those done more than 10 years ago, in other languages, containing simple fistula management, and Crohn’s fistula treatment only.

Quality Assessment Tools

We used Assessing the Methodological Quality of Systematic Reviews (AMSTAR) for systematic review and meta-analysis, Cochrane risk of bias for clinical trials, the Newcastle-Ottawa scale for cohort studies, and a scale for the quality assessment of narrative review articles (SANRA) for traditional reviews. Studies of low quality were excluded.

Data Collection

Data collection and quality assessment were done by two independent assessors. We collected the following data from the studies: first author’s name, name of the journal and publication year, type/types of procedures, number of patients included in the study, outcome/conclusion of the study, and type of research. Disagreements were resolved by discussion.

Results

**Figure 1:**
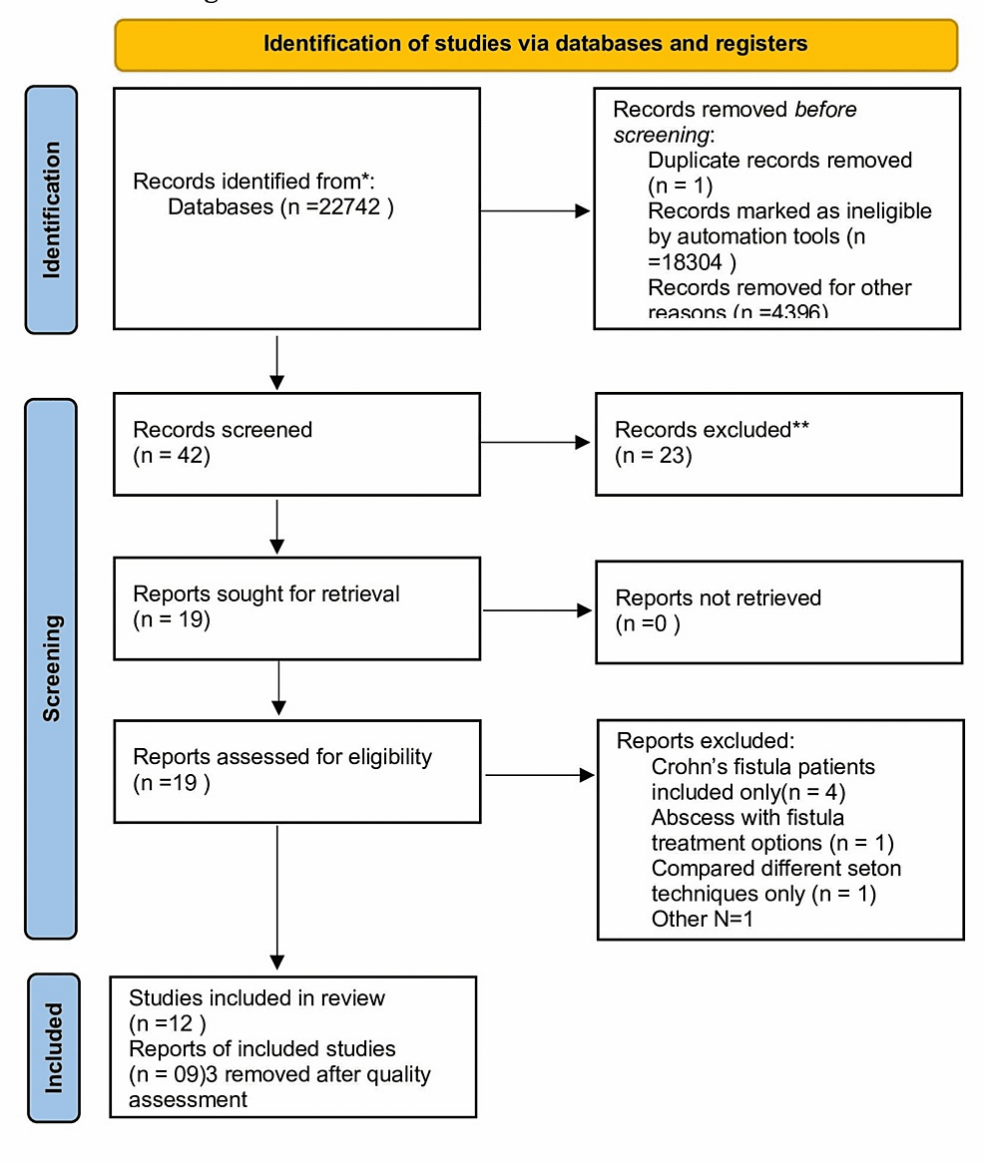
PRISMA flow diagram

After searching the database, we collected a total of 42 articles, of which 38 were from PubMed and four were from Google Scholar. After screening the titles and abstract, we selected 19 articles. Of these, we kept 12 articles and excluded the rest by applying our exclusion criteria as they were not relevant. Twelve articles were assessed for quality, of which only nine were included and the rest were excluded because they were not of good quality. Figure 1 shows the PRISMA diagram.

Table 1 shows the characteristics of the included studies.

**Table 1:** Study characteristics FP: fistula plug, MAF: mucosal advancement flap, FG: fibrin glue, AF: advancement flap (anodermal flap or MAF), ASC: autologous stem cells, IFA: island flap anoplasty, RWAF: rectal wall advancement flap, LIFT: ligation of the intersphincteric fistula tract, SR: sphincter reconstruction, ABx: antibiotics, SPS: sphincter-preserving seton, K-seton: Kashaarasootra seton

Author	Journal and publication year	Procedures	Number of patients included	Outcome/conclusion	Type of research
Göttgens et al. [13]	Springer-Verlag Berlin Heidelberg, 2014	Seton versus FG, AF versus AF+FG, MAF versus MAF+gentamicin, ASC versus ASC+FG versus FG+placebo, IFA versus seton, K-seton versus fistulectomy, RWAF versus MAF, LIFT versus MAF, MAF versus fistulotomy+sphincter reconstruction, FG+ABx versus FG+surgical closure versus FG+ABx+surgical closure, SPS versus seton, FP versus MAF (2009), FP versus MAF (2011), FP versus MAF (2010)	802	No single method found to be the most effective	Systematic review and meta-analysis
Maternini et al. [14]	Asian journal of surgery, 2020	Salvecoll-E gel injection	70	Salvecoll-E gel is a promising noninvasive technique	Cohort study
Abdelnaby et al. [15]	International Journal of Surgery (London, England), 2019	Drained mucosal advancement flap versus rerouting seton	97	Both techniques found effective (drained mucosal advancement flap versus rerouting seton)	Randomized trial
Jayne et al. [16]	Health Technology Assessment (Winchester, England), 2019	Anal fistula plug versus surgeon’s preference for transsphincteric fistula-in-ano (advancement flap, cutting seton, fistulotomy, and LIFT)	304	Fistula plug not preferable and economical as compared to other groups	Randomized trial
Scoglio et al. [17]	Clinics in Colon and Rectal Surgery, 2014	Biologic mesh, fibrin glue, fistula plug, bio-LIFT, stem cells	1,156	Biologic alternatives typically should be considered low-risk options with variable success rates	Traditional review
Vergara-Fernandez et al. [18]	World Journal of Gastroenterology, 2013	LIFT	592	LIFT procedure is an effective surgical technique and has a low impact on fecal continence	Traditional review
Schulze et al. [19]	Springer-Verlag Italia Srl, 2014	Seton drainage plus partial fistulotomy and subsequent ligation of intersphincteric fistula tract	75	Low recurrence rate after performing LIFT as a staged procedure following seton insertion and partial fistulotomy of any tract lateral to the sphincter complex	Prospective cohort study
Limura et al. [20]	World Journal of Gastroenterology, 2015	LIFT, anal fistula plug, fibrin glue, fibrin laser closure, video-assisted anal fistula treatment, adipose-derived stem cells	1,125	The best intervention is the one adapted to the individual fistula	Traditional review
O’Riordan et al. [21]	Diseases of the Colon & Rectum, 2012	Anal fistula plug insertion	530	Fistula closure by using the anal fistula plug is approximately 54% in patients without Crohn’s disease	Systematic review

Discussion

A complex anal fistula is a challenging problem in which treatment of anorectal sepsis results in some disturbance of the continence state. A 2014 systematic review and meta-analysis including 14 RCTs concluded that no single technique is most effective and that mucosal advancement flap (MAF) remains the most investigated technique [13]. The study stated that due to a small number of RCTs, it is challenging to compare all currently available techniques. The meta-analysis compared two techniques only: fistula plug (FP) and MAF. There were no differences in recurrence and complication rates. The quality of life and continence levels were not different, but it cannot be compared in this meta-analysis because of different measurement tools. The three RCTs compared in the study showed significant differences in inclusion criteria, methods, and postoperative management [13]. The differences make the result of the meta-analysis less convincing, as other nonrandomized studies showed less favorable results for the FP. One RCT compared LIFT and mucosal advancement flap (MAF). All patients included in the RCT had prior insertion of seton for six months to reduce sepsis. However, a small number of patients were included in the trial study, i.e., 25 in the LIFT group and 14 in the MAF group, and for the LIFT group, the follow-up was not as long as the MAF group (16.4 months versus 30 months, respectively) [13]. No significant variance was seen in the rate of recurrence, with 8% and 4% recurrence, respectively [13]. No variations were seen in preoperative and postoperative continence. Patients with LIFT were more satisfied with less postoperative pain scores and early return to usual activities.

The Salvecoll-E gel is indigenous collagen that is deantigenated and purified, non-cross-linked equine dermal extract; it has an amino acid composition similar to human collagen [14]. As the gel is injected into the dermis or other tissues that are damaged, fibroblasts and other nonresident cells start to mobilize from the surrounding tissues and invade the collagen gel. Moreover, the injection fills the defect immediately, and a transition matrix forms at the injection site (aseptic inflammation), which results in the stimulation of the immune system and the activation of macrophages, fibroblasts, and granulocytes associated with increased transport of growth factors, which are released from cells, which leads to accelerated migration and proliferation of epithelial cells and fibroblasts. The gel provides a matrix for the cells, which results in new tissue formation. Fibroblasts form new collagen fibers at the site of injection, and collagenase enzymes produced by fibroblasts degrade the gel slowly, which is replaced by the collagen synthesized in the body. Salvecoll-E gel injection in the fistula-in-ano tract is a new method for fistula repair that has no effect on continence. Salvecoll-E gel injection (non-cross-linked equine collagen) in a 2020 cohort study, after the removal of loose seton insertion, showed healing of 78.5% after 12 months and a recurrence rate of 21.5% [14].

An RCT of 97 patients comparing rerouting seton versus drained mucosal advancement flap found both techniques effective with a success rate of 10%, which is lower than other sphincter-sparing techniques for complex anal fistula [15]. In this study, mucosal advancement flap was done along with rerouting seton around the external anal sphincter (EAS), and the seton was cut before it divided through sphincter muscles. This technique was associated with reduced fecal incontinence levels, but operative and healing times were prolonged as compared to rerouting seton around the internal anal sphincter (IAS). These techniques may not be suitable for all complex anal fistulas as for recurrent fistula-in-ano, the IAS may have been divided in previous surgery or the intersphincteric space may have been obliterated by fibrosis and scarring. Also, the mucosa can be tough and not suitable for flap mobilization and construction in recurrent fistulas or fistulas with significant intersphincteric sepsis [15]. The author concluded that these techniques can add more options for the surgeon but need further study at large centers to reproduce the results.

An RCT published in 2019 compared the fistula plug and surgeon’s preference groups for transsphincteric fistula-in-ano (advancement flap, cutting seton, fistulotomy, and LIFT) and found that the healing rates were 54% and 55%, respectively, at the 12-month follow-up [16]. Overall, 304 participants were added to the trial. Fistula healing rates were 54 out of 110 (49%) participants in the fistula plug group and 63 out of 112 (56%) participants in the surgeon’s preference group using MRI [16]. At 12 months, the clinical healing rates were quite variable depending on the type of surgical procedure performed: fistula plug, 55%; cutting seton, 64%; fistulotomy, 75%; advancement flap, 53%; and LIFT procedure, 42% [16]. Complications were common in both groups. The only significant difference between the two groups was a higher rate of pain in the fistula plug group, which is unexpected as no tissue dissection was done. Complications due to treatment include extrusion of fistula plug (16%), extrusion of cutting seton (18%), wound complications due to fistulotomy (15%), advancement flap complications (18%), and wound complications related to LIFT (15%) [16]. Re-interventions were similarly frequent, and the majority of re-interventions were surgical rather than medical care.

Biologic alternatives (biological mesh, fibrin glue, fistula plug, and stem cells) are low-risk options, but their success rates are quite variable. The main advantage is minimal impact on continence, but reliable, long-term quantitative and qualitative data from large trials are required. Newer techniques should be evaluated critically until reproducible results are achieved to reach the desired goals of low recurrence, improved quality of life, and preservation of continence [17]. The combination of LIFT with biological materials produces improved results as compared to LIFT alone in one of the studies done in 2012.

A review published in 2013 stated that the LIFT procedure is effective for transsphincteric fistulas mainly with a fistula closure rate of 74.6% and has a small effect on continence, and the outcomes were better at the first surgical attempt [18]. The use of a bioprosthetic mesh (Bio-LIFT procedure) reported a success rate of 94%, and the use of a plug (LIFT-PLUG procedure) resulted in a success rate of 95% [18], but the Bio-LIFT technique requires extensive dissection in intersphincteric plane, and the bioprosthetic materials used are costly. None of the studies described any incontinence after LIFT, but recurrence rates were variable in each study. Also, it is less effective in recurrent fistulas. The author recommends the use of an endorectal advancement flap (ERAF) for recurrent fistulas. The combined use of biological material with LIFT requires more study as not enough evidence is available to recommend combining these two.

A prospective cohort study published in 2014 managed complex anal fistula with seton drainage plus partial fistulotomy followed by ligation of the intersphincteric fistula tract. This study reported a success rate of 88%. The use of a draining seton before the definitive procedure is becoming common in the treatment of anorectal fistulas [19]. In 2007, the anal fistula plug consensus advised using seton temporarily to remove the infection before inserting a fistula plug [19]. The LIFT procedure should be delayed in the presence of active infection in the anorectal area; instead, insertion of a draining seton should be done for a maximum of 6-8 weeks before LIFT, but waiting for longer periods results in a more difficult dissection at the time of LIFT procedure [19].

A review published in 2015 included the study of LIFT, anal fistula plug, fibrin glue, fistula laser closure, video-assisted anal fistula treatment, and adipose-derived stem cells. Fourteen studies were included in the review of LIFT, which showed healing rates ranging from 47% to 100%, but the follow-up period was variable in all studies, ranging from two weeks to 26 weeks [20]. Anal fistula plug studies showed variable results but has no negative impact on continence. Fibrin glue showed poor results; fistula laser closure does not affect continence, but as it is a blind procedure, there is a high chance of recurrence, and it requires expensive equipment as compared to other techniques. Adipose-derived stem cell technique has limited data available to date to make a judgment. This study concluded that to improve the healing rates, patient selection is vital, and controlling sepsis and identifying secondary extensions and fistula tracts from the anal canal before attempting any repair are strongly recommended [20].

Fistula closure using the fistula plug technique was found to be 54% in a study published in 2012 in non-Crohn’s disease patients [21]. The anal fistula plug (AFP) is a good option in the management of transsphincteric fistula-in-ano in patients in which the risk of incontinence associated with fistulotomy is very high [21]. However, the success rates reported vary considerably from as low as 20% to as high as 86%, and this leaves the surgeon unclear on how to state it to the patient while doing preoperative counseling [21].

The poor healing after the surgical treatment of transsphincteric fistula-in-ano demands that further research is needed to understand the pathophysiology of this common disease. LIFT seems to be a promising new technique with improved outcomes as compared to other procedures according to the data gathered in this study. Limited data is available on the treatment of complex fistula, especially randomized trials comparing different methods. Our future recommendation is to have randomized trials comparing multiple methods to see the efficacy, especially comparing LIFT with other procedures, including a large number of patients, and standardizing the inclusion and exclusion criteria, and have the same follow-up periods to compare healing, incontinence, and recurrence rates.

Limitations of the Study

The small number of studies available comparing different surgical techniques for treating complex anal fistula makes it difficult to find one effective method.

## Conclusions

A complex anal fistula is quite common yet poorly treated and is a cause of poor quality of life in the population affected. Unfortunately, no single technique has been recognized as a gold standard for treating complex anal fistula. In this article, we tried to identify a single best technique. In the past few years, alternative techniques such as biological materials and Salvecoll-E gel injection have also been introduced, which showed good results while preserving continence, but whether they are beneficial in preventing recurrence and are superior to the surgical options still remains questionable. LIFT seems to be a promising technique that preserves continence but with variable recurrence rates in different studies. In addition, the criteria comparing effectiveness were quite variable, and follow-up times and patient selection were different, which makes it difficult to compare the results. More studies and RCTs are needed to be done to compare LIFT with other procedures regarding the outcomes of recurrence and fecal incontinence to find the most effective surgical treatment for this chronic and recurrent condition.

Given the higher recurrence rates, we do not have a good understanding of the disease process, which warrants further research to be undertaken to improve quality of life. According to the data gathered in this study, LIFT is the only surgical technique that causes no incontinence, so it can be used alone and in combination with other techniques to provide better results. Trials are required with longer follow-up periods to see and understand the recurrence rates better for this technique. Whether to combine it with seton insertion beforehand depends on the type of fistula and the individual patient. Biologic alternative use is not widespread at the moment, but there is hope in the future that they might develop as a nonsurgical treatment for fistula.
